# Allegheny County Medical Society

**Published:** 1888-11

**Authors:** W. M. Brinton

**Affiliations:** President, in the Chair


### ALLEGHENY COUNTY MEDICAL SOCIETY.

W. M. BRINTON, M. D., PRESIDENT, IN THE CHAIR.

SPECIAL MEETING, SEPT. 18, 1888.

Dr. J. M. Batten reported a case of typhoid fever due to the escape of sewer gas into the bedroom from defective plumbing of a bath tub.

Dr. J. J. Green reported a case of scarlet fever because of the apparent mystery as regards the source of the contagion that surrounded it. The patient was three and a half years old, and died on the fourth day of the disease. It developed a temperature of 105°, the rash and sore throat. The child had not been from home—had not been away from the house for a month or two. Inquiry was made at the health office as to whether any case had been reported from that neighborhood; on investigation, it was found none had been reported.

Dr. Thomas: I presume the gentleman does not mean to have us understand the case began *de novo*, or without contagion from somewhere. Very often it is a very difficult matter to discover how children contract contagious diseases, but by following the cases through our practice, we may often learn how children at a distance are exposed to the diseases or their germs. For instance, last summer I attended a boy of eight years, a marked case of scarlet fever. He had a sister somewhat younger. This case ran its course before the sister took the disease. I had ceased my visits, probably about ten days, when I was called again to see the sister. I made inquiry as to the condition of the boy, and learned he was convalescent; he had been out in the street cars every day in order to give him fresh air. It is possible that the disease may have been acquired in the street car by some other child. Again, a mother may have been sitting for twenty minutes beside that convalescent and the germ may have contaminated her clothing.

Dr. Batten: I believe that there are many cases that originate spontaneously. I remember several years ago I attended a couple of cases of scarlet fever; the children, all that were in the family, died with the disease. In about ten years after that time (the mother having in the meantime borne two or three other children) the children then living were attacked with scarlet fever. Immediately after the death of the first children with the disease, the rooms in which they had died were papered. Before the second lot of children were attacked with scarlet fever, they tore off the paper and re-papered the room. There were no cases of scarlet fever in the neighborhood at that time, and the only cause I could assign for the origin of these cases of scarlet fever was the tearing of the paper from the room in which the first children died. We all know that the germs of scarlet fever can be sent in letters, and in so many different ways that it is hardly strange that we have some cases of spontaneous scarlet fever.

Dr. Lange: It seems to me that Dr. Batten argues very lucidly that spontaneity in these cases is out of the question. In the cases referred to by Dr. Batten, the germs covered by the wall-paper were uncovered and thus freed to become the cause of the fever. His illustration of the disease germs being carried by letter also points to the conclusion that scarlet fever cannot originate *de novo*. We know that scarlet fever depends upon a germ which must always be received by the patient taking the fever, although we are very often unable to discover in what manner the patient received it. Still, Dr. Batten explains his cases in a very admirable manner and argues, in fact, that there is a specific germ which is necessary to the development of the fever; that it cannot develop unless the patient has received this germ, although the means by which he has received it may be very obscure and impossible to discover.

Dr. W. P. Munn read a paper on

CHRONIC INTESTINAL OBSTRUCTION—REPORT OF CASE AND OPERATION. (SEE DECEMBER NUMBER.)

Dr. Thomas said that he had no doubt that many cases of intestinal obstruction perished, as this one would probably have done, for lack of operation. He related the case of a lad on whom he performed abdominal section ten years ago, failing, however, to find the origin of the trouble. The patient died of peritonitis. He believed that in the present state of knowledge and antiseptic practice the child might have been saved.

Dr. Buchanan suggested that in Dr. Munn's case the mere fact of the presence of small cysts in the second ovary could hardly be considered sufficient justification for its removal; that the tendency now was rather toward the preservation of such ovaries.

Dr. J. C. Lange related a case of pleuritis in which aspiration of the chest was immediately followed by the death of the patient. The patient, a man aged forty-seven years, of about five feet four inches in height, weighing about 180 pounds, presented a right side acute pleuritis, and suffered from an unusually great dyspnœa. All the signs of considerable impairment of circulation and oxygenation existed, with a frequent and feeble pulse. The needle was entered between the fifth and sixth ribs in the axillary line, the patient being in a reclining position. After seventeen ounces of the effusion, which was large, had entered the aspiration bottle, the patient expired. A post mortem was not had. Presumably the heart was fatty; if so, it may have been stilled by the slight shock of the operation.

A CASE OF IMPERFORATE ANUS

was reported by Dr. J. D. Thomas. Female infant presented absence of urethra and anus. An opening from the bladder and one from the rectum into the vagina were discovered by the probe. Under anæsthesia, an opening was established in the perineum; a bent probe was passed into the rectum through the vagina and was the guide for the knife. The child died. I think it well to wait for perhaps forty-eight hours or more in these cases to see if nature will press down the rectum, and in that way give an idea as to where it is located.

Dr. Buchanan: About two years ago I saw, with Dr. Christie, of Allegheny, a case of imperforate anus which was operated on. It was a male child, born the night before. There was no sign of an anus at all. Dr. Christie passed a bistoury up in front of the sacrum, and after it had entered about an inch, some meconium appeared, and he then enlarged the opening with his finger. The child did well and is living to-day.

Dr. Hulseton: I had two cases of that kind occurring in my practice in Allegheny, one of them a male child, perfectly developed in every other respect. There was no indication of an anus or a place for an anus. I operated on this case immediately, making every possible effort to find the gut. Could not introduce the smallest probe.

---

THE HOAGLAND LABORATORY.—The trustees of the Hoagland Laboratory take pleasure in announcing to the medical profession of Brooklyn the completion of the laboratory and its equipment for practical work.

Special facilities are offered to those who desire to prosecute original research. For this purpose private laboratories have been provided, and arrangements are now being made for the purchase of a library which shall contain all the literature necessary for reference in the departments of Bacteriology, Physiology and Pathology.

The fee for this course of instruction has been placed at $15, which will entitle the subscriber to prosecute his studies until June 1, 1889, during as many hours of the day as he may desire.

